# Drug dependence in patients with chronic pain: A retrospective study

**DOI:** 10.1097/MD.0000000000012748

**Published:** 2018-10-05

**Authors:** Tomoko Tetsunaga, Tomonori Tetsunaga, Keiichiro Nishida, Hirotaka Kanzaki, Haruo Misawa, Tomoyuki Takigawa, Yasuyuki Shiozaki, Toshifumi Ozaki

**Affiliations:** a Department of Orthopaedic Surgery, Okayama University Hospital, Okayama; b Department of Orthopaedic Surgery, Kurashiki Municipal Hospital, Kurashiki; c Department of Pharmacy, Okayama University Hospital, Okayama, Japan.

**Keywords:** chronic pain, drug dependence, pain catastrophizing

## Abstract

Drug dependence, which can exist concurrently with chronic pain, is seen as one of the major causes of rapidly increasing medical expenses. However, drug dependence in patients with chronic pain has not been evaluated. The aim of this study was to identify the risk factors for drug dependence in patients with chronic noncancer pain.

This retrospective study included 151 patients with chronic noncancer pain (43 males, 108 females; mean age, 72 years). Low back pain (LBP) occurred in 96 patients, whereas 22 had shoulder pain, 8 had hip pain, and 77 had knee pain. Patients were divided into drug dependence and nondrug dependence groups based on the Severity of Dependence Scale (SDS) scores. Patients with SDS scores ≥5 and <5 were classified into drug dependence and nondrug dependence groups, respectively. All patients completed self-report questionnaires. Factors that predict drug dependence were identified by performing univariate and multivariate analyses.

Sixty (40%) of the 151 patients met the SDS criteria for drug dependence. Significant differences were found between patients with and without drug dependence for the LBP, hip pain, number of medications, and for the Numerical Rating Scale, Pain Disability Assessment Scale (PDAS), Hospital Anxiety and Depression Scale, and Pain Catastrophizing Scale (PCS) scores. Multiple regression analysis identified LBP, hip pain, PCS, and PDAS scores as factors related to drug dependence in patients with chronic noncancer pain.

Drug dependence tends to differ in patients based on the location of their chronic pain. Pain catastrophizing and disability indicated a greater tendency for drug dependence. Thus, PCS and PDAS scores are useful screening tools for predicting drug dependence in patients with chronic pain.

## 1 Introduction

Although pain is a major complaint among patients visiting the hospital, it is often undertreated. Therefore, higher number of visits to health services are reported for patients who experience persistent pain and long-term disability.^[1]^ Both neuropathic and nociceptive pain mechanisms are associated with chronic low back pain (LBP).^[2,3]^ Neuropathic pain patients show higher levels of pain intensity accompanied by more comorbidities such as depression, panic/anxiety, and sleep disorders.^[3,4]^ Prolonged LBP can also be potentially be caused by psychological factors, occupational disabilities, and somatization disorders.^[5]^ As nonsteroidal anti-inflammatory drugs (NSAIDs) have a limited effect in patients with chronic neuropathic pain, it is necessary to treat these patients with other drugs such as pregabalin or tramadol/acetaminophen.^[6,7]^

Although the abuse of opioids can lead to drug dependence,^[8]^ the potential for abuse is much lower for NSAIDs, acetaminophen, and analgesic plasters. However, the undertreatment of patients with persistent chronic pain can lead to a strong desire for oral medication. Drug dependence in patients is a chronic condition of the brain that results from compulsive drug use. Even though the user is aware of the negative consequences, a lack of self-control results in the compulsive drug use. The 3 symptoms that characterize drug dependence are as follows: psychological dependence in which patients have a compulsive desire to ingest more of the drug to obtain a desired effect; physical dependence characterized by an inability to perform normal activities and functions of daily life, as well as the occurrence of withdrawal symptoms when they stop using the drug; and tolerance, in which patients need to consume increasing amounts of a drug to obtain the desired effect.^[9]^ Although opioid addiction has frequently been reported during treatments for back pain,^[10]^ to the best of our knowledge, drug dependence in patients with chronic pain has not been analyzed in detail. Therefore, the purpose of this study was to investigate drug dependence in patients with chronic noncancer pain.

## 2 Methods

The present retrospective study was performed at the authors’ institution. All patients provided written, informed consent to participate in the study. Ethical approval was obtained from our institution's Institutional Review Board. This study recruited 151 patients (43 males, 108 females) with chronic pain. Inclusion criteria included persistent pain for ≥3 months, conservative treatment for pain for ≥1 month, and the patient's agreement to complete questionnaires. Exclusion criteria included dementia, heavy users of alcohol, illicit drug users, or other conditions that would make it difficult to complete self-report written questionnaires. Patients were also excluded if they had a severe chronic disease such as cardiovascular disease or renal failure that would interfere with the treatment.

The mean age of the participants at the time of examination was 72 years (range, 25–92 years), with a mean duration of pain from the onset of symptoms of 52 months (range, 3–696 months). Table 1 shows the drugs administered during the study. There were 32 (21.2%) chronic pain patients who were only treated with NSAIDs. Patients were divided into 2 groups based on the Severity of Dependence Scale (SDS) scores (Table 2). The SDS was devised as a way to provide a short, easily administered self-report scale. This scale can examine different types of drugs and measure the degree of dependence experienced by users.^[11]^ The 5 multiple-choice items used by the SDS can be easily changed to modify references to the named drug or specific time span, respectively, thereby making it possible to cover different drugs and time frames.^[12]^ All items are explicitly concerned with the psychological components of dependence. Scores for each of the items range from 0 to 3, with a total scoring range of 0 to 15. Higher scores indicated a greater degree of dependence on the drug in question. Although earlier work with the SDS suggested that a cutoff score of 4 or 5 was indicative of dependence, this has yet to be statistically validated.^[12]^ In the present study, we judged a SDS score of 5 or more points to indicate drug dependence. In addition to the SDS score, data were collected on the patients’ demographic and clinical backgrounds.

**Table 1:**
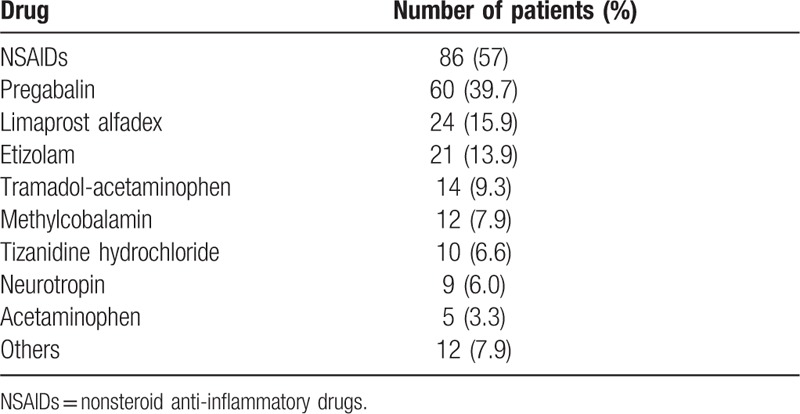
Administered medications.

**Table 2:**
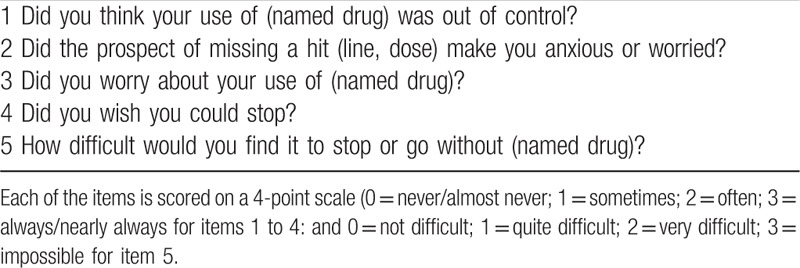
Severity of Dependence Scale (SDS).

### 2.1 Pain assessment

To measure the intensity of chronic pain, the Numeric Rating Scale (NRS) for pain self-assessment is a widely used, valid, and reliable tool. NRS scores range from 0 to 10, with 0 representing no pain and 10 representing the worst pain imaginable.

### 2.2 Physical disability assessment

The items used for the Pain Disability Assessment Scale (PDAS) are designed to assess the negative effects of pain on broad-spectrum pain interference domains.^[13]^ The 20 items used for PDAS are rated on a 4-point Likert scale, with scores ranging from 0 to 60 points. Clinicians who require a multidimensional measure of the effects of pain have used the PDAS to examine a patient's daily routine.

### 2.3 Anxiety and depression assessment

The Hospital Anxiety and Depression Scale (HADS) was used to assess anxiety and depression.^[14]^ Patients with physical illness can be easily assessed for anxiety and depression by using the HADS. The HADS is a 14-item scale, with 7 items assessing anxiety and 7 assessing depression. Each item is rated from 0 to 3 on a 4-point Likert scale. The overall scores for anxiety or depression range between 0 and 21, with higher scores indicating a greater severity of symptoms.

### 2.4 Pain catastrophizing assessment

The Pain Catastrophizing Scale (PCS) is used to assess self-reported pain catastrophizing due to chronic pain.^[15]^ The PCS, which is a broad measure of pain catastrophizing, is composed of 13 items rated from 0 (never) to 4 (always) on a 5-point Likert scale. The maximum score for the PCS is 52, with higher scores indicating greater pain catastrophizing levels. High levels of catastrophizing are defined by a score of >24. The items on the PCS are divided into the following 3 subscales: rumination, helplessness, and magnification. Rumination (items 8–11) “refers to the fact that the patient cannot get the idea of pain out of his/her head and cannot stop thinking about the pain.” Helplessness (items 1–5 and 12) “refers to the estimation that the person has not been able to do anything to influence the pain.” Magnification (items 6, 7, and 13) “refers to the exaggeration of the threatening properties of the painful stimulus.”

### 2.5 Statistical analysis

Patients with SDS scores ≥5 and <5 were classified into drug dependence and nondrug dependence groups, respectively. Univariate analyses were then conducted between the groups to compare age, sex, duration of pain, number of medications, pain complaints, and the NRS, PDAS, HADS, and PCS scores. Normally distributed variables were compared using a Student *t* test. Nonnormally distributed variables were compared using the Mann–Whitney *U* test. Chi-square analysis was performed for categorical variables. Values of *P* < .05 were considered significant. Factors predicting drug dependence were identified by multivariate analysis (multiple regression). Potential predictive variables were included in the multivariate model if values of *P* < .05 were obtained on univariate analysis. A multiple regression model and 95% confidence intervals (CIs) were used to identify the risk factors that influence drug dependence. Statistical analysis was conducted using SPSS software version 22.0 for Windows (IBM, Tokyo, Japan).

## 3 Results

### 3.1 Patients’ background

In this study, 96 patients had LBP, 22 had shoulder pain, 8 had hip pain, and 77 had knee pain. Patients were divided into 3 groups. The LBP group consisted of 50 patients with LBP only (18 males, 32 females), the Joint pain group consisted of 55 patients with joint pain only (14 males, 41 females), and the Both pain group consisted of 46 patients with both LBP and joint pain (11 males, 35 females) (Table 3). Although there was no significant difference seen for the sex among the 3 groups (*P* = .35), mean age and duration of pain were significantly higher in the Both versus the LBP group (*P* = .02 and <.01, respectively). As shown in Table 3, there was a significantly lower number of medications (*P* = .02), and significantly lower PDAS (*P* = .02), PCS (*P* < .01), HADS (*P* < .01), and SDS scores (*P* < .01) in the Joint pain group compared with the other 2 groups. However, the NRS scores were significantly higher in the Both pain group compared with the other 3 groups (*P* < .01).

**Table 3:**
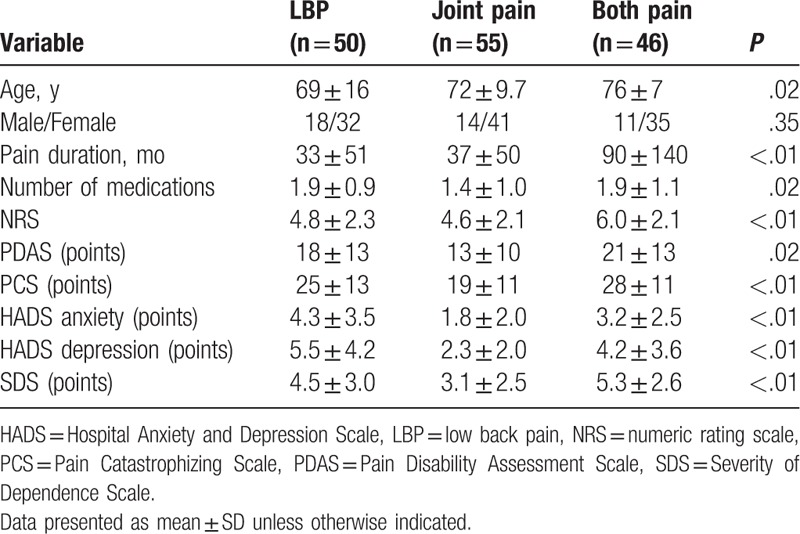
Patients’ demographics.

### 3.2 Univariate analyses with or without drug dependence

Among all of the patients, 60 (40%) met the criteria for drug dependence according to their SDS scores. The 60 patients in the drug dependence group were composed of 21 males and 39 females, whereas the 91 patients in the nondrug dependence group were composed of 22 males and 69 females. Table 4 shows the univariate analyses that examined the demographic data between the 2 groups. Significant differences were observed for the SDS scores between the groups (*P* < .01). No significant associations were found between the drug dependence and mean age (*P* = .14) and the sex (*P* = .15). Among the drugs used in this study, pregabalin was administered at significantly higher rate in patients with drug dependence than without drug dependence (*P* < .01). Significant differences were observed for all of the other variables (*P* < .01) with the exception for shoulder pain (*P* = .73) and knee pain (*P* = .84).

**Table 4:**
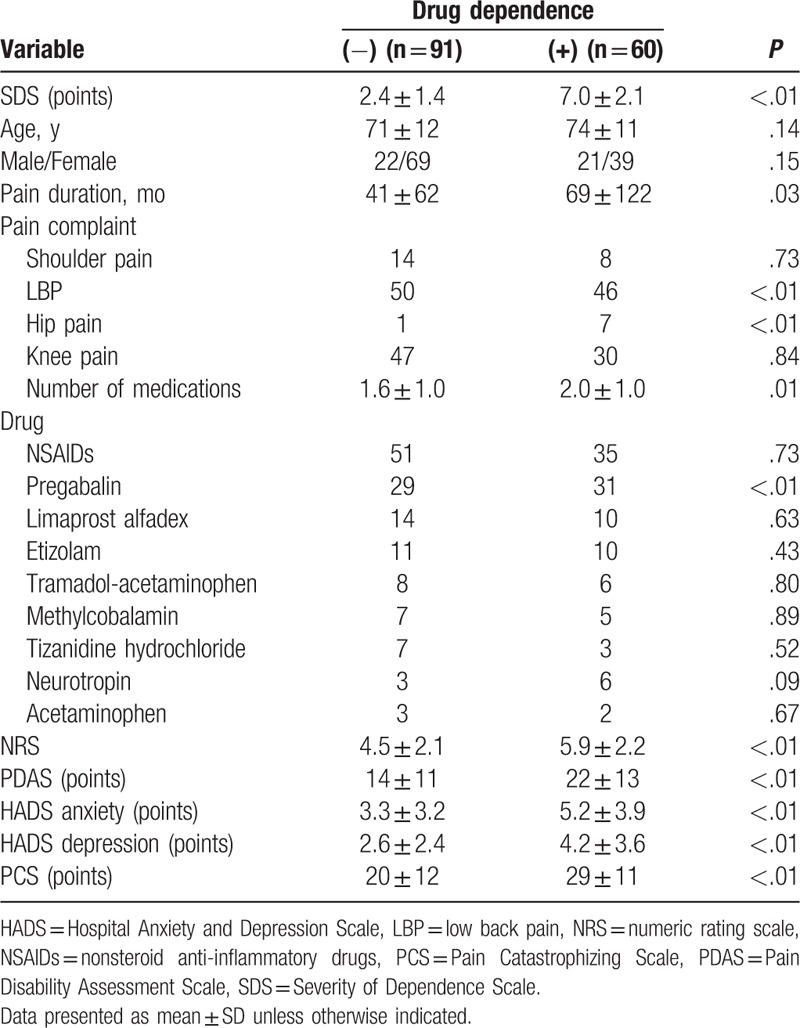
Univariate analyses with or without drug dependence.

### 3.3 Correlation between drug dependence and risk factors

The data in Table 4 suggest that SDS scores were associated with some of the risk factors. In these cases, we used a simple linear correlation to evaluate the presence of these potential correlations. Multiple regression analysis was performed to investigate the association between drug dependence and nondrug dependence, with drug dependence used as the response variable, and pain duration, number of medications, LBP, hip pain, and the NRS, PDAS, HADS, and PCS scores used as the explanatory variables. We found that the SDS scores (*y*) were positively correlated with LBP (*x*_1_), hip pain (*x*_2_), and the PCS (*x*_3_) and PDAS scores (*x*_4_) (Table 5). These results provided the following prediction formula: *y* = 1.054 + 0.987*x*_1_ + 2.717*x*_2_ + 0.055*x*_3_ + 0.062*x*_4_. The adjusted coefficient of determination was 0.317, and all *P* values were <.05, indicating that the variables chosen for analysis in this study had good explanatory power.

**Table 5:**
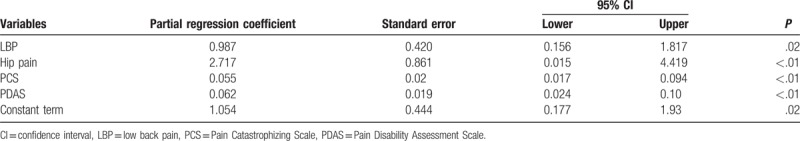
Correlation between factors and Severity of Dependence Scale (SDS).

## 4 Discussion

The results of this study revealed that drug dependence, as defined by SDS scores, was present in 40% of the patients with chronic noncancer pain. Drug dependence differed according to the site of pain, and was higher among chronic LBP patients versus chronic joint pain patients. Furthermore, among the chronic joint pain patients, drug dependence was higher in patients with hip pain than in those with pain in other joints. Multiple regression analysis revealed that high PCS and PDAS scores were associated with an increased risk of drug dependence. These findings suggest that these factors are useful indices that can be used when setting up treatments for patients with chronic noncancer pain.

When assessing drug dependence in patients being treated for chronic pain, it has been reported that heavy users of alcohol or illicit drug user need to be excluded.^[10,16]^ Thus, none of the patients included in the present study were alcohol misusers or illicit drug users. Furthermore, iatrogenic substance abuse or addiction needs to be differentiated from preexisting substance abuse or addiction. A previous study reported that the prevalence of drug dependence predating the onset of back pain was 77%.^[17]^ Another study reported that 94% of patients had substance abuse problems before the onset of their back pain.^[18]^ These reports suggest that most substance abuse or addiction problems may predate chronic back pain and related opioid treatment. Chronic pain and addiction can coexist either on a continuum or as separate comorbid conditions. In pain management, controlled substances are viewed as either a problem or a solution depending on the healthcare professional's training and perspective.^[19]^ Only a few pain training programs are known to offer significant experiential and didactic training in drug abuse and addiction, even though chronic pain and addiction often coexist.^[20]^ There is a low risk for iatrogenic abuse or addiction in patients with no previous history of substance abuse or addiction in short-term treatment for noncancer-related back pain. Even so, the undertreatment of chronic pain cannot be ethically justified in well-selected patients.^[10]^ Therefore, it is important that clinicians be able to recognize the difference between true addiction and similar conditions.^[21]^ Opioid-seeking behaviors are known to increase in patients when chronic pain is left untreated or undertreated.^[22]^ In the present study, 21.2% of the patients with chronic noncancer pain were treated with NSAIDs only. As it has been reported that NSAIDs have only limited effects in chronic pain patients, a weak opioid analgesic such as tramadol should be considered as an alternative treatment.^[7]^ However, other studies have found that the long-term efficacy of opioids for chronic pain may be limited.^[16,23]^

As most pain conditions involve various pathways, analgesic therapy using a single agent may be inadequate for relieving chronic pain. It has been suggested that more effective pain relief for a broader spectrum of pain could be achieved by using combination analgesics with 2 or more agents that have synergistic analgesic effects.^[24]^ Moreover, a decreased incidence of adverse drug reactions has been reported when a combination of individually ineffective doses of tramadol and acetaminophen are used to provide adequate pain relief through the actions of multipathways.^[24]^ However, in the absence of a clear treatment policy, this could introduce the risk of increased dosage levels.

Pincus et al examined the transition to chronic pain status and reported finding strong evidence for the role of negative mood (distress or depression).^[25]^ Psychological factors are one of the causes of this change.^[25]^ It has also been reported that depression and sleep disorders, which cause functional impairment,^[26]^ can lead to reduced pain thresholds.^[27]^ Therefore, patients receiving prolonged pain management tend to have persistent pain. The present study found that many patients with drug dependence also had depressive conditions. Thus, the results of our study suggested that the dominant factors that influenced drug dependence were pain catastrophizing and disability. Cognitive behavioral therapy has been widely accepted for use in patients with alcohol and drug dependence. We believe that multidisciplinary approaches, including physical therapy are necessary to treat patients with pain catastrophizing and disability. A recent study reported that patients treated for chronic intractable pain at a pain liaison clinic showed significant improvement in pain catastrophizing and anxiety after 6 months of treatment.^[28]^ We also believe that drug dependence is “a family illness,” as a patient's family plays a crucial role in recovery because the drug dependent patients lack insight into their disease. Therefore, cognitive behavioral therapy with the patients’ families has been recommended.^[29]^

There were some limitations for the present study. Firstly, the sample size of this study was small. This study examined drug dependence in patients with chronic noncancer pain according to different pain locations, which included the lumbar area, shoulder, hip, and knee. However, there were only 8 patients with hip pain included due to its low morbidity rate. Moreover, we analyzed which drug could induce drug dependence using univariate and multiple regression analyses. However, analyses of the 9 different drugs made it even smaller-size study. This small number of patients is considered the weakness of this study. Second, we did not classify patients based on the nature of the chronic pain. The results of this study indicated that patients with LBP were found to be more dependent to drugs. Generally, 64.7% were found to have possible neuropathic pain in an unselected cohort of chronic LBP patients.^[3]^ Patients with neuropathic pain show higher ratings of pain intensity,^[3]^ and tend to be intractable than nociceptive pain. As neuropathic and nociceptive components contribute to chronic pain, these components require different pain management strategies because of their different pathogeneses.^[4]^ There is a possibility that drug dependence could be examined in more detail by classifying chronic pain patients according to type of pain. Despite these limitations, this study does provide new knowledge on drug dependence. Patients with LBP and hip pain were at a higher risk of drug dependence versus patients with shoulder or knee pain. Trials that have tried to visualize chronic pain using functional magnetic resonance imaging (fMRI) have been actively carried out in recent years.^[30]^ The application of techniques such as fMRI and brain imaging are expected to result in further developments and improvements in identifying and treating patients with drug dependence. In addition, based on the correlation we found for the PCS and PDAS scores with drug dependence, these scores might be beneficial screening tools that can be used at the time of the first examination in order to prevent drug dependence. The results of this study could very well lead to improvements in the treatment of patients with chronic pain.

## Author contributions

**Conceptualization:** Tomoko Tetsunaga, Tomonori Tetsunaga, Keiichiro Nishida, Hirotaka Kanzaki, Toshifumi Ozaki.

**Data curation:** Tomoko Tetsunaga.

**Formal analysis:** Tomoko Tetsunaga, Tomonori Tetsunaga.

**Investigation:** Tomoko Tetsunaga.

**Methodology:** Tomoko Tetsunaga, Tomonori Tetsunaga, Keiichiro Nishida, Hirotaka Kanzaki, Tomoyuki Takigawa, Yasuyuki Shiozaki, Toshifumi Ozaki.

**Supervision:** Tomonori Tetsunaga, Haruo Misawa, Tomoyuki Takigawa, Yasuyuki Shiozaki.

**Writing—original draft:** Tomoko Tetsunaga, Tomonori Tetsunaga.

**Writing—review and editing:** Tomoko Tetsunaga, Tomonori Tetsunaga, Keiichiro Nishida, Hirotaka Kanzaki, Haruo Misawa, Tomoyuki Takigawa, Yasuyuki Shiozaki, Toshifumi Ozaki.
